# Synthesis and Biological Evaluation of Norcantharidin Derivatives Possessing an Aromatic Amine Moiety as Antifungal Agents

**DOI:** 10.3390/molecules201219782

**Published:** 2015-12-02

**Authors:** Yang Wang, Wenbo Sun, Shunqing Zha, Huan Wang, Yalin Zhang

**Affiliations:** 1 State Key Laboratory of Crop Stress Biology for Arid Areas, College of Plant Protection, Northwest A & F University, Yangling 712100, China; wangyang2006@nwsuaf.edu.cn (Y.W.); zsq2623@163.com (S.Z.); 2 Key Laboratory of Plant Protection Resources and Pest Management, Ministry of Education, Northwest A & F University, Yangling 712100, China; wbsun06@163.com (W.S.); huanwang1107@gmail.com (H.W.)

**Keywords:** cantharidin, natural product, structural modification, arylamine, fungicidal activity

## Abstract

Based on the structure of naturally produced cantharidin, different arylamine groups were linked to the norcantharidin scaffold to provide thirty six compounds. Their structures were confirmed by melting point, ^1^H-NMR, ^13^C-NMR and HRMS-ESI studies. These synthetic compounds were tested as fungistatic agents against eight phytopathogenic fungi using the mycelium growth rate method. Of these thirty six derivatives, seven displayed stronger antifungal activity than did norcantharidin, seven showed higher activity than did cantharidin and three exhibited more significant activity than that of thiabendazole. In particular, 3-(3′-chloro-phenyl)carbamoyl norcantharidate **II-8** showed the most significant fungicidal activity against *Sclerotinia fructigena* and *S. sclerotiorum*, with IC_50_ values of 0.88 and 0.97 μg/mL, respectively. The preliminary structure-activity relationship data of these compounds revealed that: (1) the benzene ring is critical for the improvement of the spectrum of antifungal activity (3-phenylcarbamoyl norcantharidate **II-1** vs norcantharidin and cantharidin); (2) among the three sites, including the C-2′, C-3′ and C-4′ positions of the phenyl ring, the presence of a halogen atom at the C-3′position of the benzene ring caused the most significant increase in antifungal activity; (3) compounds with strongly electron-drawing or electron-donating groups substitutions were found to have a poor antifungal activity; and (4) compared with fluorine, bromine and iodine, chlorine substituted at the C-3′ position of the benzene ring most greatly promoted fungistatic activity. Thus, compound **II-8** has emerged as new lead structure for the development of new fungicides.

## 1. Introduction

Fungal pathogens are the primary causes of both plant diseases and postharvest losses [1,2], and thus contribute to severe damage to global crop production [3]. To guard against fungal pathogens, one traditional approach is to employ synthetic fungicides, which are both economical and efficient, and have played an indispensable role in nourishing more people throughout human history. Unfortunately, drug resistance, environmental hazards and many other drawbacks have emerged along with this fungicide utilization [4,5]. This requires that novel antifungal agents continue to be discovered. It is well-known that insect secondary metabolites result from their adaptation to the environments during the long period of evolution in insects, such as for defense against predation or infection, and pesticides produced from insect secondary metabolites may result in less or slower resistance development and lower pollution [6]. Hence, insecticides of natural source have been considered as attractive alternatives to synthetic agrochemicals for pest management [7]. Bio-based insecticides, such as nereistoxin, nicotine, pyrethrum, and neem extracts, are made by organisms as defense against insects [8].

**Figure 1:**
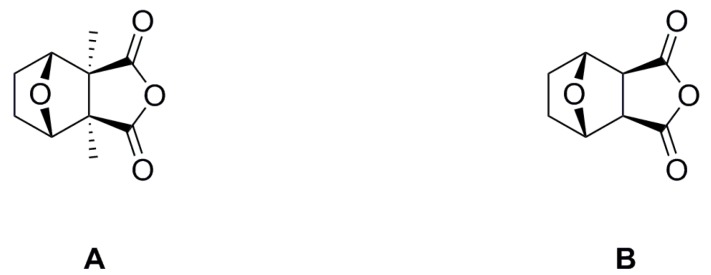
The structures of cantharidin (**A**) and norcantharidin (**B**).

Cantharidin (CTD, Figure 1A), a naturally occurring terpene, is the main secondary metabolite isolated from the bodies of the blister beetles, including *Mylabris cichorii*, *M. phalerata* and *Epicauta chinensis* [9,10]. Besides its use as the lead compound for the preparation of potent anticancer drugs, such as its analogues sodium cantharidinate and norcantharidin [11,12,13,14], CTD also shows interesting antifungal and insecticidal activities [15,16,17,18].

Norcantharidin (NCTD, Figure 1B), a demethylated analogue of CTD, is well known as a strong inhibitor of protein serine/threonine phosphatases (PSPs) [19], a broad class of PSPs associated with signaling and control of numerous cellular processes in many organisms [20]. The biological activities associated with NCTD are derived from its abilities to inhibit the family of PSPs. Furthermore, the catalytic domain of all PSP subfamilies is highly conserved in insects, plants, phytopathogenic fungi and all the other eukaryotes [21]. NCTD, as the strong inhibitor, binds to a hydrophobic pocket of the PSP active site [22]. The structural similarity between CTD and NCTD has been apparent to animal scientists and similar mechanisms of action on animals have been confirmed [23]. Previous studies by our group demonstrated that both *in vivo* and *in vitro*, there are significant inhibitory effects from CTD, NCTD and their analogues on PSPs of *Plutella xylostella*, suggesting that their modes of action may be related to impeding insect PSPs activity [24]. More recently, strong evidence indicates that the commercial herbicide mode of action of endothall, an analogue of NCTD, was intimately related to the inhibition of PSPs [25].

**Figure 2:**
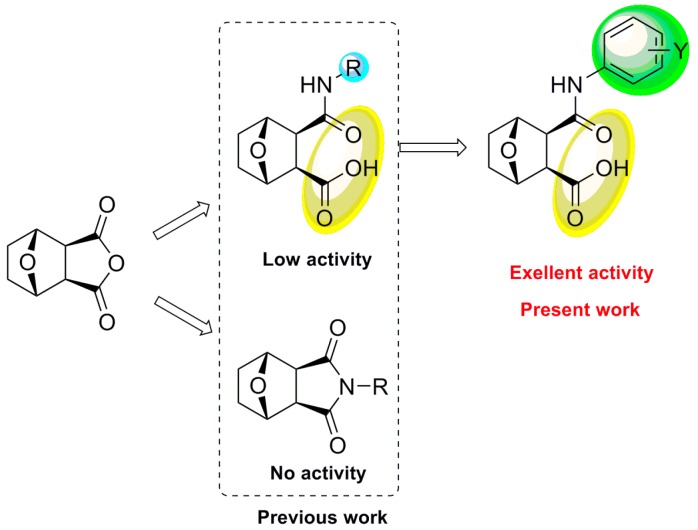
Design of the title compounds.

Meanwhile, as illustrated in Figure 2, our previous investigation on the structure-activity relationship (SAR) of NCTD and its anhydride modified derivatives against *P. xylostella* indicated that the structures of oxygen bridge and carboxyl were essential for the biological activity, and that the improvement of bioactivity required a reasonable R group, the combination of both aliphatic amide and aromatic amide moieties. And the type of substituent Y, substituted on the phenyl ring, was also critical for the improvement of insecticidal activity [26].

**Scheme 1:**
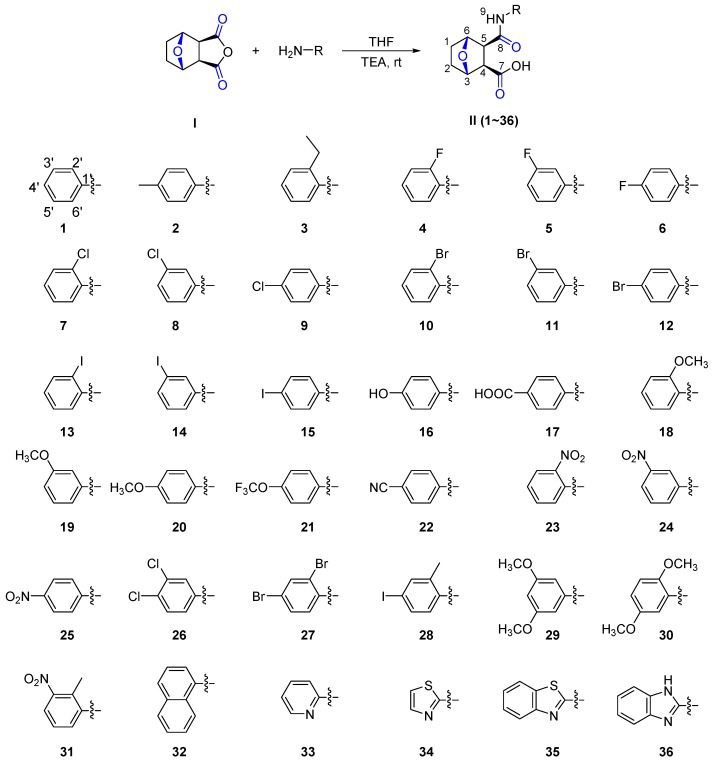
The Synthesis and Structures of Compounds **II** (**1**–**36**).

To date, few attempts have been made to develop a biorational fungicide from NCTD via chemical modification. Encouraged by the abovementioned results and in continuation of our program aimed at the discovery and development of natural-product-based fungicidal agents, in this study, thirty six aromatic amine derivatives of NCTD modified in the anhydride ring were synthesized (Scheme 1), and their biological activities against eight phytopathogenic fungi were evaluated using the mycelium growth rate method [27]. Additionally, their preliminary SAR studies were also described.

## 2. Results and Discussion

### 2.1. Chemistry

As shown in Scheme 1, the target compounds **II** were synthesized by the aminolysis reaction of NCTD **I** and various halogen aromatic amines in the presence of triethylamine as the binding acid agent, and synthesized via decomposing the anhydride ring of NCTD **I** by a halogenated aniline with different electronegativity [28].

### 2.2. Antifungal Activity

Preliminary *in vitro* screening results of the title compounds for antifungal activities against eight fungi at the concentration of 50 μg/mL are listed in Table 1.

**Table 1:** Preliminary antifungal activities of compounds at 50 μg/mL.

Compd.	Values of Inhibition Rate (%) to Eight Pathogens							
	*Valsa mali*	*Botryosphaeria berengeriana*	*Sclerotinia fructigena*	*Glomerella cingulate*	*Alternaria alternate*	*Sclerotinia sclerotiorum*	*Alternaria solani*	*Cochliobolus sativum*
**II-1**	68.42 ± 1.99d *	68.64 ± 0.65e	72.20 ± 0.66e	62.35 ± 0.99f	65.52 ± 1.06de	76.39 ± 0.92e	61.10 ± 0.96e	67.01 ± 0.59c
**II-2**	0.66 ± 0.06q	1.27 ± 0.03qr	0.53 ± 0.06qr	5.38 ± 0.05tu	9.66 ± 0.06no	23.61 ± 0.06p	1.52 ± 0.10u	7.98 ± 0.06qr
**II-3**	18.42 ± 0.06mn	11.02 ± 0.10l	18.72 ± 0.06m	6.99 ± 0.06st	11.72 ± 0.06n	20.17 ± 0.00q	1.01 ± 0.06u	10.11 ± 0.06pqr
**II-4**	74.99 ± 1.30c	69.90 ± 1.17e	76.48 ± 1.63d	80.66 ± 1.18d	70.34 ± 1.41c	86.69 ± 0.85d	76.77 ± 0.26c	66.50 ± 0.30c
**II-5**	83.56 ± 1.05b	87.70 ± 0.91c	85.03 ± 1.79bc	90.34 ± 2.64b	84.82 ± 1.30a	92.27 ± 0.06c	83.35 ± 1.16b	81.39 ± 0.38b
**II-6**	41.45 ± 0.47e	39.39 ± 1.61f	49.21 ± 1.37f	55.40 ± 1.36g	41.38 ± 2.14g	46.34 ± 2.29f	47.46 ± 1.21f	51.59 ± 0.76e
**II-7**	34.21 ± 1.00f	22.86 ± 1.85h	45.47 ± 2.26g	24.74 ± 0.83h	38.62 ± 1.04g	35.19 ± 1.78g	30.29 ± 0.92g	35.06 ± 1.80f
**II-8**	86.85 ± 1.00a	91.54 ± 1.33b	100.00 ± 0.00a	92.96 ± 2.69a	86.90 ± 2.32a	100.00 ± 0.00a	89.90 ± 0.79a	91.50 ± 0.66a
**II-9**	32.88 ± 3.85f	22.01 ± 2.89h	28.36 ± 2.69h	24.18 ± 1.08h	30.34 ± 3.13h	26.60 ± 2.79i	28.27 ± 1.83hi	17.53 ± 0.90j
**II-10**	25.01 ± 3.06g	18.18 ± 3.79i	23.00 ± 2.51i	21.48 ± 1.28i	23.44 ± 2.27i	30.46 ± 3.04h	18.17 ± 2.45j	21.21 ± 2.53i
**II-11**	80.92 ± 1.26b	81.77 ± 0.90d	83.42 ± 2.49c	84.97 ± 1.47c	78.63 ± 2.28b	96.13 ± 1.32b	79.83 ± 1.81bc	83.56 ± 1.80b
**II-12**	20.39 ± 1.04h	15.24 ± 3.23i	17.13 ± 2.59j	18.25 ± 1.37j	22.07 ± 3.14i	20.59 ± 2.45j	25.23 ± 1.87i	13.78 ± 1.91k
**II-13**	15.14 ± 1.23i	10.99 ± 2.47j	22.46 ± 1.62i	12.35 ± 0.59k	17.22 ± 4.17j	15.87 ± 1.37k	16.16 ± 2.31j	9.54 ± 1.22l
**II-14**	65.79 ± 1.00d	66.51 ± 1.95e	70.60 ± 2.19e	60.77 ± 0.78f	62.76 ± 2.13e	73.80 ± 3.43e	58.60 ± 1.33e	63.86 ± 1.01d
**II-15**	3.29 ± 1.16j	−5.12 ± 3.86k	1.07 ± 0.92k	5.37 ± 0.86l	3.44 ± 1.18k	8.15 ± 1.43l	11.10 ± 6.18k	−2.71 ± 1.98m
**II-16**	9.21 ± 0.10p	0.85 ± 0.04pqr	8.56 ± 0.06p	9.14 ± 0.03rs	10.34 ± 0.06no	6.87 ± 0.06t	2.02 ± 0.10u	5.85 ± 0.10s
**II-17**	0.66 ± 0.06q	0.42 ± 0.06qr	−0.53 ± 0.06r	0.54 ± 0.06wx	1.38 ± 0.06pq	0.43 ± 0.08u	7.58 ± 0.12s	12.23 ± 0.10nopq
**II-18**	1.97 ± 0.06q	6.36 ± 0.06mn	0.00 ± 0.00qr	0.00 ± 0.00x	8.28 ± 0.06no	0.00 ± 0.06u	0.00 ± 0.00u	0.00 ± 0.00t
**II-19**	0.66 ± 0.06q	10.17 ± 0.06l	2.67 ± 0.06qr	11.83 ± 0.05pq	15.86 ± 0.06m	0.00 ± 0.06u	2.53 ± 0.00u	11.70 ± 0.06opq
**II-20**	0.66 ± 0.06q	2.12 ± 0.10pqr	0.53 ± 0.06qr	0.00 ± 0.00x	2.07 ± 0.06pq	0.00 ± 0.06u	0.00 ± 0.00u	0.00 ± 0.00t
**II-21**	1.32 ± 0.10q	10.59 ± 0.06l	11.76 ± 0.10o	15.59 ± 0.06o	20.69 ± 0.06ijk	13.30 ± 0.06s	21.72 ± 0.06lm	22.87 ± 0.06hi
**II-22**	24.34 ± 0.06k	0.00 ± 0.066r	8.02 ± 0.11p	12.90 ± 0.05p	17.93 ± 0.06jklm	18.45 ± 0.06q	22.73 ± 0.06kl	12.23 ± 0.03nopq
**II-23**	19.74 ± 0.06m	0.00 ± 0.00r	9.09 ± 0.11p	3.23 ± 0.03uv	0.69 ± 0.10pq	13.73 ± 0.10t	2.53 ± 0.03u	11.17 ± 0.06opq
**II-24**	25.00 ± 0.10q	5.08 ± 0.06n	18.18 ± 0.10m	20.43 ± 0.06m	28.28 ± 0.06h	19.31 ± 0.06q	7.07 ± 0.03st	15.43 ± 0.10lm
**II-25**	23.03 ± 0.10kl	2.54 ± 0.11pq	12.30 ± 0.06o	17.20 ± 0.05no	21.38 ± 0.10ij	7.73 ± 0.06u	3.54 ± 0.03tu	6.38 ± 0.11rs
**II-26**	32.89 ± 0.10j	18.22 ± 0.06k	48.13 ± 0.06g	28.49 ± 0.06h	35.86 ± 0.10g	40.34 ± 0.06k	33.33 ± 0.12op	37.23 ± 0.06f
**II-27**	36.18 ± 0.06hi	15.25 ± 0.06j	39.57 ± 0.06i	22.04 ± 0.06jkl	20.69 ± 0.06ijk	42.96 ± 0.06j	29.80 ± 0.06ij	14.89 ± 0.06lmn
**II-28**	8.55 ± 0.06p	6.78 ± 0.06o	39.04 ± 0.06i	23.66 ± 0.06ijk	38.62 ± 0.10fg	42.06 ± 0.00jk	16.67 ± 0.10nop	12.77 ± 0.06mnop
**II-29**	30.26 ± 0.06j	0.42 ± 0.06qr	36.36 ± 0.06j	21.51 ± 0.06kl	40.00 ± 0.06f	36.48 ± 0.06l	20.20 ± 0.06lmn	12.77 ± 0.10mnop
**II-30**	0.66 ± 0.06q	0.00 ± 0.06r	14.97 ± 0.10n	4.30 ± 0.06tuv	10.34 ± 0.16no	26.18 ± 0.06no	12.12 ± 0.10qr	18.62 ± 0.10jk
**II-31**	15.13 ± 0.10o	0.00 ± 0.06r	17.11 ± 0.06m	4.84 ± 0.00tuv	-1.38 ± 0.06q	12.02 ± 0.06s	7.07 ± 0.10st	12.23 ± 0.11nopq
**II-32**	3.29 ± 0.10 q	8.05 ± 0.06m	6.42 ± 0.06p	5.91 ± 0.06tu	6.90 ± 0.06o	7.30 ± 0.00t	18.69 ± 0.06mno	22.34 ± 0.06hi
**II-33**	0.66 ± 0.06q	0.00 ± 0.11r	28.88 ± 0.06k	2.69 ± 0.06vw	-0.69 ± 0.05q	23.61 ± 0.08p	6.57 ± 0.06st	−1.60 ± 0.07t
**II-34**	36.84 ± 0.10h	0.42 ± 0.06qr	30.48 ± 0.06k	0.54 ± 0.06x	0.69 ± 0.06pq	69.53 ± 0.06h	13.13 ± 0.06pqr	15.43 ± 0.10lm
**II-35**	30.26 ± 0.15g	2.97 ± 0.06o	21.39 ± 0.10l	5.91 ± 0.06tu	0.00 ± 0.03pq	0.00 ± 0.00u	0.51 ± 0.06u	10.11 ± 0.03pqr
**II-36**	61.93 ± 0.10e	72.88 ± 0.06e	68.98 ± 0.15f	62.37 ± 0.06e	61.38 ± 0.03d	77.25 ± 0.06f	62.63 ± 0.06e	65.43 ± 0.03cd
Norcantharidin	88.17 ± 1.87a	34.31 ± 1.69g	86.64 ± 0.80b	17.72 ± 1.21j	20.00 ± 1.10ij	24.46 ± 1.13i	1.98 ± 2.24l	29.75 ± 1.27g
Cantharidin	88.16 ± 1.39a	38.54 ± 2.68f	100.00 ± 0.00a	10.19 ± 1.61k	67.59 ± 1.06cd	84.54 ± 1.39d	70.20 ± 0.84d	68.05 ± 1.29c
Thiabendazole	86.84 ± 1.22a	100.00 ± 0.00a	100.0 ± 0.00a	66.67 ± 0.00e	47.58 ± 1.60f	100.00 ± 0.00a	15.14 ± 1.18j	24.99 ± 1.17h

* The differences between data with different letters within a column are significant for the same tested fungus (*p* < 0.05) with respect to TBZ, cantharidin, norcantharidin and these synthetic compounds with a halogenated benzene moiety.

The results in Table 1 indicate that seven of the synthetic derivatives displayed significant activities (61.1%–100% inhibitory rate) against all eight tested fungi at a concentration of 50 µg/mL. On the contrary, another twenty nine derivatives showed lower activity at the same concentration. Here, the inhibition rates were for only given as IC_50_ values for further comparison.

**Figure 3:**
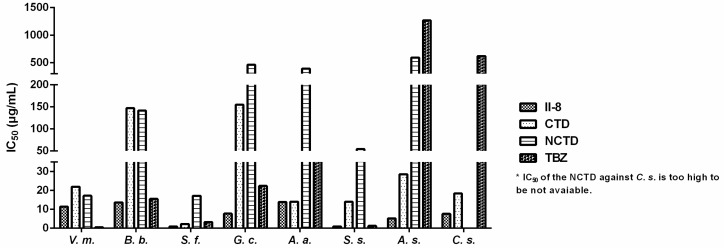
IC_50_ of compound **II-8**, CTD, NCTD and TBZ against the eight tested fungi.

Encouraged by these preliminary findings, we planned further SAR studies on the title compounds. We determined their IC_50_ values by the mycelial growth inhibitory rate method. As shown in Table 2, the tested compounds presented different fungicidal activity against the eight plant pathogenic fungi, superior to the corresponding parent compound NCTD in some cases, and they were the same as or more active than thiabendazole (TBZ) against some of the tested fungi. Of these compounds, 3-(3′-chlorophenyl)carbamoyl norcantharidate **II-8** exhibited the most significant activity on all the eight fungi. As illustrated in Figure 3, compound **II-8** showed much better activity than that of TBZ, NCTD and CTD. Notably, compound **II-8** showed excellent antifungal properties against *Sclerotinia fructigena* and S. *sclerotiorum*, with IC_50_ values of 0.88 and 0.97 μg/mL, respectively.

### 2.3. SAR

#### 2.3.1. Effect of Introducing the Benzene Ring on Fungistatic Activity

Illustrated in Table 2, NCTD, without a benzene ring, exhibited low fungistatic activity at the concentration of 50 μg/mL against the tested strains. Introducing one benzene (**2a**) caused a significant increase in fungistatic activity against the plant-pathogenic fungi *Botryosphaeria berengeriana*, *S. fructigena*, *Glomerella cingulate*, *Alternaria alternate*, *S. sclerotiorum*, *A. solani* and *Cochliobolus sativum*, with IC_50_ values of 31.2568, 14.6778, 36.3526, 42.2537, 7.2482, 30.1335 and 22.7788 μg/mL, respectively. At the same time, as seen in Table 2, compared with the fungicidal activities of CTD against *B. berengeriana* and *G. cingulate*, the compound **II-1** also showed almost 5-fold more fungistatic activity than CTD, respectively. On the contrary, the introduction of a heterocyclic ring gave us a negative contribution to fungistatic activity.

#### 2.3.2. Effect of Position Substituted on the Benzene Ring on Fungistatic Activity

Substituting a halogen at the C-3′position of the benzene ring (*i.e*., compounds **II-5**, **II-8** and **II-11**) improved the fungicidal activity in some cases. It was also clear that the site substituted on the phenyl ring plays an important role in activity, as compounds substituted at the C-4′ position (**II-6**, **II-9** and **II-12**) and C-2′ position (**II-4**, **II-7** and **II-10**) were all found to be less active than their analogues.

**Table 2:** Antifungal activity of some compounds against eight pathogens *.

Compd.	IC50 (μg/mL) (CI 95%) **							
	*V. m.*	*B. b.*	*S. f.*	*G. c.*	*A. a.*	*S. s.*	*A. s.*	*C. s.*
**II-1**	32.0800 (26.1510–39.3533)	31.2568 (23.7127–41.2012)	14.6778 (10.7872–19.9716)	36.3526 (29.1302–45.3658)	42.2537 (33.7577–52.8879)	7.2482 (2.9912–17.5637)	30.1335 (23.3387–38.9065)	22.7788 (16.0894–32.2493)
**II-4**	27.8016 (22.1312–34.9249)	24.8446 (18.2585–33.8063)	10.6665 (7.6148–14.9412)	16.7764 (11.7587–23.9352)	31.4258 (24.6239–40.1066)	4.2858 (1.6648–11.0334)	14.0938 (9.1043–21.8178)	10.4051 (12.9130–24.7109)
**II-5**	20.1058 (14.8666–27.1914)	19.8375 (14.7821–26.6217)	2.0791 (1.3098–3.3003)	11.2505 (6.9283–18.2691)	18.1002 (13.1268–24.9579)	3.4556 (1.6958–7.0418)	10.4753 (6.7490–16.2590)	10.4051 (6.4109–16.8880)
**II-8**	11.3756 (7.2790–17.7778)	13.6528 (9.8721–18.8814)	0.8805 (0.4243–1.8275)	7.7364 (3.8335–15.6128)	13.8916 (9.3377–20.6664)	0.9698 (0.4790–1.9633)	5.1863 (2.5133–10.7020)	7.5908 (4.4564–12.9298)
**II-11**	17.0896 (11.9965–23.3450)	17.0640 (12.6223–23.0689)	2.3717 (1.4367–3.9152)	9.1071 (5.1797–16.0125)	22.1465 (16.7792–29.2308)	2.1783 (0.9142–5.1904)	12.1081 (7.9591–18.4200)	12.9298 (8.9552–18.6685)
**II-14**	47.7810 (39.0992–58.39.4)	37.3895 (28.8394–48.4746)	21.9666 (16.3757–29.4664)	57.0963 (45.2452–72.0517)	49.1546 (39.1730–61.6795)	9.6192 (4.2886–21.5754)	34.8290 (27.5093–44.0964)	29.8409 (16.0894–32.2493)
Norcantharidin	17.1862 (9.2272–32.0104)	141.8133 (61.1287–328.9946)	17.1673 (10.0952–29.1937)	465.1719 (86.5242–2500.8598)	394.9566 (73.9654–2108.9689)	54.5899 (32.0147–93.0839)	594.2606 (211.1064–1672.8327)	NA ***
Cantharidin	21.9320 (13.3453–36.0436)	147.4225 (46.8714–463.6810)	2.2175 (0.6116–12.0751)	155.0286 (76.7546–313.1261)	14.0428 (5.6729–34.7622)	14.0622 (8.0013–24.7142)	28.5223 (18.6015–43.7340)	18.3900 (8.3854–40.3309)
TBZ	0.5191 (0.0218–12.3413)	15.5378 (8.8501–27.2793)	3.1998 (1.2133–8.4391)	22.3806 (10.4428–47.9653)	47.8696 21.2217–107.9790	1.2947 (0.6661–2.5165)	1268.5889 (110.6555–1453.4930)	621.7874 60.1464–6427.9804

* *V. m.*: *Valsa mali*, *B. b*.: *Botryosphaeria berengeriana*, *S. f.*: *Sclerotinia fructigena*, *G. c.*: *Glomerella cingulate*, *A. a.*: *Alternaria alternate*, *S. s.*: *Sclerotinia sclerotiorum*, *A. s.*: *Alternaria solani*, *C. s.*: *Cochliobolus sativum*. ** CI 95%: Confidence interval at 95% probability (µg/mL). *** NA: Not Available.

#### 2.3.3. Effect of Various Substituents on the Benzene Ring on Fungistatic Activity

The presence of different halogen atoms on the benzene ring resulted in various effects on fungistatic activity against the eight fungi tested. As can be seen from Table 2, at the C-3′position of the benzene ring, introduction of the chlorine atom (**II-8**) produced a more significant increase in the fungistatic activity against all eight fungi tested than introductions of a fluorine atom (**II-5**) or a bromine atom (**II-11**). In contrast, the introduction of an iodine atom on the benzene ring (**II-14**) resulted in a negative influence on fungistatic activity compared with compound **II-1** with no substitution on the phenyl ring.

Compounds with substitutions of strongly electron-drawing (CN, compound **II-22**; NO_2_, compounds **II-23**, **24**, **25**) or electron-donating (CH_3_, compound **II-2**; CH_2_CH_3_, compound **II-3**; OCH_3_, compounds **II-18**, **19**, **20**) groups were found to have a poor antifungal activity. In order to draw firm conclusions about the significance of these data, compounds containing one phenyl ring substituted by two methoxy groups in different positions (compounds **II-29**, **30**) were also tested, and showed the same poor activity. The nitro group was tested in other positions in combination with a methyl group (compound **II-31**), but only poor spectrum and antifungal activity were detected. Further aromatic groups were also evaluated. Compounds with the phenyl moiety replaced with several kinds of heterocycle produced relatively poor levels of antifungal activity compared to the corresponding compound **II-1**.

## 3. Experiment Section

### 3.1. General Information

Cantharidin (CTD, ≥98%) was isolated from *M. phalerata* (Chinese blister beetle), bought from the Chinese herbal medicine market in Xian, China. Norcantharidin (NCTD, ≥98%) was purchased from Alfa Aesar Chemical Co. Ltd., (Haverhill, MA, USA). Dimethyl sulfoxide (DMSO, ≥99%) was obtained from J & K China Chemical Ltd. (Beijing, China). The fungicide thiabendazole (TBZ, ≥99.1%) was purchased from Sigma-Aldrich Trading Co. Ltd. (Shanghai, China). All reagents and solvents were of reagent grade or purified according to standard methods before use. Analytical thin-layer chromatography (TLC) was performed with silica gel plates using silica gel 60 GF_254_ (Qingdao Haiyang Chemical Co., Ltd., Qingdao, China). Silica gel column chromatography was performed with 200–300 mesh silica gel (Qingdao Haiyang Chemical Co., Ltd.).

Melting points were determined on a WRS-2 apparatus equipped with a microcomputer (Shanghai Precision & Scientific Instrument Co. Ltd., Shanghai, China) and are uncorrected. Proton nuclear magnetic resonance (^1^H-NMR) spectra and carbon nuclear magnetic resonance (^13^C-NMR) spectra were recorded at 500 and 125 MHz, respectively, on a Bruker Avance III 500 MHz NMR spectrometer (Karlsruhe, Germany) in DMSO-*d*_6_ using tetramethylsilane (TMS) as the internal standard. High-resolution mass spectrometry (HRMS) was carried out using a Bruker micrOTOF focus II instrument.

### 3.2. Synthesis of the Title Compounds ***II(1–36)***

To a solution of NCTD (**1**, 1.0 g, 5.95 mmol) dissolved in tetrahydrofuran (THF, 10 mL) accompanied by triethylamine (TEA, 0.5 mL) as the acid-binding agent was added the corresponding aromatic amine (1 equiv., 5.95 mmol). When the reaction was complete after 14 h as checked by TLC analysis, the solution was concentrated under reduced pressure and diluted with acetone (100 mL). The resulting filter cake was either recrystallized from methanol or purified by column chromatography (MeOH/CH_2_Cl_2_, 1: 4, *v*/*v*) to afford the desired products **II** (**1**–**36**). The yields, physical properties, ^1^H-NMR, ^13^C-NMR, and HRMS-ESI of the target compounds **II** were as follows:

*3-(Phenylcarbamoyl)-7-oxabicyclo[2.2.1]heptane-2-carboxylic acid* (**II-1**). Yield 76%, white solid, mp: 164–165 °C; ^1^H-NMR δ: 1.47–1.68 (m, 4H, H-1, 2), 2.93–3.12 (m, 2H, H-4, 5), 4.61–4.81 (m, 2H, H-3, 6) 7.03 (t, *J* = 7.41 Hz, 1H, H-4′), 7.29 (t, *J* = 7.72 Hz, 2H, H-3′, 5′), 7.54 (d, *J* = 7.88 Hz, 2H, H-2′, 6′), 9.67 (s, 1H, H-9), 11.99 (br, s, 1H, H-10); ^13^C-NMR δ: 28.91 (C-2), 29.46 (C-1,), 52.09 (C-4), 53.98 (C-5), 77.33 (C-3), 79.20 (C-6), 123.41 (C-2′, C-6′), 127.28 (C-4′), 129.06 (C-3′, C-5′), 139.75 (C-1′), 169.78 (C-8), 172.73 (C-7); HR-MS (ESI): *m*/*z* calcd. For C_14_H_15_NO_4_Na ([M + Na]^+^) 284.0899, found 284.0910.

*3-(p-Tolylcarbamoyl)-7-oxabicyclo[2.2.1]heptane-2-carboxylic acid* (**II-2**). Yield 58%, white solid, mp: 211–212 °C; ^1^H-NMR δ: 1.44–1.70 (m, 4H, H-1, 2), 2.25 (s, 3H, H-7′), 2.94 (d, *J* = 9.77 Hz, 1H, H-4), 3.05 (d, *J* = 9.46 Hz, 1H, H-5), 4.64 (d, *J* = 4.41 Hz, 1H, H-6), 4.79 (d, *J* = 3.78 Hz, 1H, H-3), 7.09 (d, *J* = 8.20 Hz, 2H, H-3′, 5′), 7.42 (d, *J* = 8.20 Hz, 2H, H-2′, 6′) 9.56 (s, 1H, H-9) 11.95 (br, s, 1H, H-10); ^13^C-NMR δ: 20.90 (s, 1C, C-7′), 28.90 (s, 1C, C-1), 29.46 (s, 1C, C-2), 52.07 (s, 1C, C-4), 53.97 (s, 1C, C-5), 77.31 (s, 1C, C-6), 79.22 (s, 1C, C-3), 119.68 (s, 2C, C-2′, C-6′), 129.41 (s, 2C, C-3′, C5′), 132.28 (s, 1C, C-1′), 137.24 (s, 1C, C-4′), 169.54 (s, 1C, C-8), 172.72 (s, 1C, C-7); HR-MS (ESI): *m*/*z* calcd for C_15_H_17_NO_4_Na ([M + Na]^+^) 298.1055, found 298.1051.

*3-((2-Ethylphenyl)carbamoyl)-7-oxabicyclo[2.2.1]heptane-2-carboxylic acid* (**II-3**). Yield 80%, white solid, mp: 172–173 °C; ^1^H-NMR δ: 1.15 (t, *J* = 7.41 Hz, 3H, H-8′), 1.54–1.73 (m, 4H, H-1, 2), 2.58 (q, *J* = 7.57 Hz, 2H, H-7′), 3.06–3.15 (m, 2H, H-4, 5), 4.74 (d, *J* = 5.04 Hz, 1H, H-6), 4.87 (d, *J* = 3.47 Hz, 1H, H-3), 7.05–7.10 (m, 1H, H-4′), 7.13–7.22 (m, 2H, H-3′, 5′), 7.66 (d, *J* = 7.88 Hz, 1H, H-6′), 8.76 (s, 1H, H-9), 12.21 (br, s, 1H, H-10); ^13^C-NMR δ: 14.61 (C-8′), 24.04 (C-7′), 28.94 (C-1, C-2), 52.26 (C-4), 54.60 (C-5), 77.56 (C-3), 79.58 (C-6), 123.85 (C-6′), 124.98 (C-3′), 126.37 (C-5′), 128.84 (C-4′), 135.71 (C-2′), 136.30 (C-1′), 169.93 (C-8), 172.82 (C-7); HR-MS (ESI): *m*/*z* calcd for C_16_H_18_NO_4_ ([M − 1]^−^) 288.1236, found 288.1246.

*3-((2-Fluorophenyl)carbamoyl)-7-oxabicyclo[2.2.1]heptane-2-carboxylic acid* (**II-4**). Yield 85%, white solid, mp: 196–197 °C; ^1^H-NMR δ: 1.53–1.68 (m, 4H, H-1, 2), 3.04 (d, *J* = 9.46 Hz, 1H, H-4), 3.21 (d, *J* = 9.77 Hz, 1H, H-5), 4.61–4.77 (m, 1H, H-6), 4.78–4.93 (m, 1H, H-3), 7.06–7.19 (m, 2H, H-3′, 5′), 7.20–7.28 (m, 1H, H-4′), 8.02 (t, *J* = 7.72 Hz, 1H, H-6′), 9.28 (s, 1H, H-9), 12.13 (br, s, 1H, H-10); ^13^C-NMR δ: 28.92 (s, 1C, C-1), 29.11 (s, 1C, C-2), 52.28 (s, 1C, C-4), 54.13 (s, 1C, C-5), 77.54 (s, 1C, C-3), 79.36 (s, 1C, C-6), 115.53 (s, 1C, C-3′), 123.24 (s, 1C, C-1′), 124.83 (s, 1C, 6′), 126.95 (s, 1C, 4′), 152.24 (s, 1C, C-5′), 154.17 (s, 1C, C-2′), 170.22 (s, 1C, C-8), 172.71 (s, 1C, C-7); HR-MS (ESI): *m*/*z* calcd. For C_14_H_14_FNO_4_Na ([M + Na]^+^) 302.0805, found 302.0807.

*3-((3-Fluorophenyl)carbamoyl)-7-oxabicyclo[2.2.1]heptane-2-carboxylic acid* (**II-5**). Yield 86%, white solid, mp: 165–167 °C; ^1^H-NMR δ: 1.41–1.71 (m, 4H, H-1, 2), 2.97 (d, *J* = 9.46 Hz, 1H, H-4), 3.07 (d, *J* = 9.46 Hz, 1H, H-5), 4.66 (d, *J* = 4.41 Hz, 1H, H-6), 4.80 (d, *J* = 3.78 Hz, 1H, H-3), 6.85 (td, *J* = 8.43, 2.36 Hz, 1H, H-4′), 7.22 (d, *J* = 8.20 Hz, 1H, H-6′), 7.29–7.36 (m, 1H, H-5′), 7.57 (d, *J* = 11.66 Hz, 1H, H-2′), 9.95 (s, 1H, H-9) 12.01 (s, 1H, H-10); ^13^C-NMR δ: 28.89 (s, 1C, C-1), 29.44 (s, 1C, C-2), 52.17 (s, 1C, C-4), 53.89 (s, 1C, C-5), 77.38 (s, 1C, C-3), 79.07 (s, 1C, C-6), 106.28 (s, 1C, C-4′), 109.72 (s, 1C, C-2′), 115.31 (s, 1C, C-6′), 130.62 (s, 1C, C-5′), 141.46 (s, 1C, C-1′), 161.65 (s, 1C, C-3′), 170.19 (s, 1C, C-8), 172.64 (s, 1C, C-7); HR-MS (ESI): *m*/*z* calcd. For C_14_H_14_FNO_4_Na ([M + Na]^+^) 302.0805, found 302.0811.

*3-((4-Fluorophenyl)carbamoyl)-7-oxabicyclo[2.2.1]heptane-2-carboxylic acid* (**II-6**). Yield 78%, white solid, mp: 186–187 °C; ^1^H-NMR δ: 1.45–1.69 (m, 4H, H-1, 2), 2.93–2.98 (m, 1H, H-4), 3.02–3.08 (m, 1H, H-5), 4.65 (d, *J* = 4.10 Hz, 1H, H-6), 4.79 (d, *J* = 3.78 Hz, 1H, H-3), 7.13 (t, *J* = 8.83 Hz, 2H, H-2′, 6′), 7.55 (dd, *J* = 8.83, 5.04 Hz, 2H, H-3′, 5′), 9.73 (s, 1H, H-8), 11.98 (br, s, 1H, H-7); ^13^C-NMR δ: 28.90 (s, 1C, C-1), 29.46 (s, 1C, C-2), 52.15 (s, 1C, C-4), 53.83 (s, 1C, C-5), 77.34 (s, 1C, C-3), 79.10 (s, 2C, C-6), 115.49 (s, 2C, C-3′, C-5′), 121.37 (s, 2C, C-2′, C-6′), 136.13 (s, 1C, C-1′), 157.37 (s, 1C, C-4′), 169.71 (s, 1C, C-8), 172.68 (s, 1C, C-7); HR-MS (ESI): *m*/*z* calcd. For C_14_H_14_FNO_4_Na ([M + Na]^+^) 302.0805, found 302.0813.

*3-((2-Chlorophenyl)carbamoyl)-7-oxabicyclo[2.2.1]heptane-2-carboxylic acid* (**II-7**). Yield 71%, white solid, mp: 177–178 °C; ^1^H-NMR δ: 1.54–1.75 (m, 4H, H-1, 2), 3.15 (q, *J* = 9.77 Hz, 2H, H-4, 5), 4.78 (d, *J* = 4.73 Hz, 1H, H-6), 4.91 (d, *J* = 3.47 Hz, 1H, H-3), 7.12 (t, *J* = 7.72 Hz, 1H, H-4′), 7.32 (t, *J* = 7.72 Hz, 1H, H-5′), 7.48 (d, *J* = 7.88 Hz, 1H, H-3′), 8.05 (d, *J* = 7.88 Hz, 1H, H-6′), 9.07 (s, 1 H, H-9), 12.30 (br, s, 1H, H-10); ^13^C-NMR δ: 28.80 (s, 1C, C-1), 28.97 (s, 1C, C-2), 52.43 (s, 1C, C-4), 54.87 (s, 1C, C-5), 77.68 (s, 1C, C-3), 79.49 (s, 1C, C-6), 123.34 (s, 1C, C-6′), 123.95 (s, 1C, C-5′), 125.36 (s, 1C, C-2′), 127.93 (s, 1C, C-3′), 129.64 (s, 1C, C-4′), 135.54 (s, 1C, C-1′), 170.25 (s, 1C, C-8), 172.61 (s, 1C, C-7); HR-MS (ESI): *m*/*z* calcd. For C_14_H_14_ClNO_4_Na ([M + Na]^+^) 318.0509, found 318.0513.

*3-((3-Chlorophenyl)carbamoyl)-7-oxabicyclo[2.2.1]heptane-2-carboxylic acid* (**II-8**). Yield 55%, white solid, mp: 178–179 °C; ^1^H-NMR δ: 1.44–1.70 (m, 4H, H-1, 2), 2.93–3.01 (m, 1H, H-4), 3.03-3.13 (m, 1H, H-5), 4.66 (d, *J* = 4.41 Hz, 1H, H-6), 4.79 (d, *J* = 3.78 Hz, 1H, H-3), 6.99–7.15 (m, 1H, H-4′), 7.23–7.43 (m, 2H, H-5′, 6′), 7.81 (s, 1 H, H-2′), 9.93 (s, 1H, H-9), 12.01 (s, 1H, H-10); ^13^C-NMR δ: 28.89 (s, 1C, C-1), 29.44 (s, 1C, C-2), 52.20 (s, 1C, C-4), 53.84 (s, 1C, C-5), 77.38 (s, 1C, C-3), 79.03 (s, 1C, C-6), 117.97 (s, 1C, C-6′), 119.17 (s, 1C, C-2′), 123.09 (s, 1C, C-4′), 130.75 (s, 1C, C-5′), 133.44 (s, 1C, C-3′), 141.18 (s, 1C, C-1′), 170.21 (s, 1C, C-8), 172.63 (s, 1C, C-7); HR-MS (ESI): *m*/*z* calcd. For C_14_H_14_ClNO_4_Na ([M + Na]^+^) 318.0509, found 318.0515.

*3-((4-Chlorophenyl)carbamoyl)-7-oxabicyclo[2.2.1]heptane-2-carboxylic acid* (**II-9**). Yield 56%, white solid, mp: 178–181 °C; ^1^H-NMR δ: 1.44–1.69 (m, 4H, H-1, 2), 2.97 (d, *J* = 9.46 Hz, 1H, H-4), 3.06 (d, *J* = 9.46 Hz, 1H, H-5), 4.66 (d, *J* = 4.41 Hz, 1H, H-6), 4.79 (d, *J* = 3.47 Hz, 1H, H-3), 7.35 (d, *J* = 8.51 Hz, 2H, H-3′, 5′), 7.57 (d, *J* = 8.83 Hz, 2H, H-2′, 6′), 9.85 (s, 1H, H-8), 12.01 (s, 1H. H-7); ^13^C-NMR δ: 28.89 (s, 1C, C-1), 29.45 (s, 1C, C-2), 52.16 (s, 1C, C-4), 53.87 (s, 1C, C-5), 77.36 (s, 1C, C-3), 79.08 (s, 1C, C-6), 121.16 (s, 2C, C-2′, C-6′), 126.93 (s, 1C, C-4′), 128.96 (s, 2C, C-3′, C-5′), 138.73 (s, 1C, C-1′), 169.96 (s, 1C, C-8), 172.66 (s, 1C, C-7); HR-MS (ESI): *m*/*z* calcd. For C_14_H_14_ClNO_4_Na ([M + Na]^+^) 318.0509, found 318.0519.

*3-((2-Bromophenyl)carbamoyl)-7-oxabicyclo[2.2.1]heptane-2-carboxylic acid* (**II-10**). Yield 45%, white solid, mp: 169–171 °C; ^1^H-NMR δ: 1.55–1.73 (m, 4H, H-1, 2), 3.14 (s, 2H, H-4, 5), 4.79 (d, *J* = 4.73 Hz, 1H, H-6), 4.91 (d, *J* = 2.84 Hz, 1H, H-3), 7.06 (t, *J* = 7.57 Hz, 1H, H-4′), 7.30–7.40 (m, 1H, H-5′), 7.63 (d, *J* = 7.88 Hz, 1H, H-3′), 7.99 (d, *J* = 7.88 Hz, 1H, H-6′), 8.99 (s, 1H, H-9′), 12.29 (br, s, 1H, H-10); ^13^C-NMR δ: 28.80 (s, 1C, C-1), 28.99 (s, 1C, C-2), 52.41 (s, 1C, C-4), 54.89 (s, 1C, C-5), 77.67 (s, 1C, C-3), 79.45 (s, 1C, C-6), 114.90 (s, 1C, C-2′), 123.93 (s, 1C, C-6′), 125.99 (s, 1C, C-5′), 128.46 (s, 1C, C-3′), 132.89 (s, 1C, C-4′), 136.77 (s, 1C, C-1′), 170.24 (s, 1C, C-8), 172.58 (s, 1C, C-7); HR-MS (ESI): *m*/*z* calcd. For C_14_H_14_BrNO_4_Na ([M + Na]^+^) 362.0004, found 362.0008.

*3-((3-Bromophenyl)carbamoyl)-7-oxabicyclo[2.2.1]heptane-2-carboxylic acid* (**II-11**). Yield 56%, white solid, mp: 189–191 °C; ^1^H-NMR δ: 1.45–1.70 (m, 4H, H-1, 2), 2.94–3.01 (m, 1H, H-4), 3.03–3.09 (m, 1H, H-5), 4.67 (br, s, 1H, H-6), 4.79 (br, s, 1H, H-3), 7.19–7.30 (m, 2H, H-5′, 6′), 7.39 (d, *J* = 7.88 Hz, 1H, H-4′), 7.96 (br, s, 1H, H-2′), 9.91 (br, s, 1H, H-9), 12.01 (s, 1H, H-10); ^13^C-NMR δ: 28.90 (s, 1C, C-1), 29.44 (s, 1C, C-2), 52.21 (s, 1C, C-4), 53.84 (s, 1C, C-5), 77.40 (s, 1C, C-3), 79.03 (s, 1C, C-6), 118.36 (s, 1C, C-3′), 121.94 (s, 1C, C-6′), 122.05 (s, 1C, C-2′), 125.99 (s, 1C, C-4′), 131.06 (s, 1C, C-5′), 141.32 (s, 1C, C-1′), 170.19 (s, 1C, C-8), 172.63 (s, 1C, C-7); HR-MS (ESI): *m*/*z* calcd. For C_14_H_14_BrNO_4_Na ([M + Na]^+^) 362.0004, found 362.0011.

*3-((4-Bromophenyl)carbamoyl)-7-oxabicyclo[2.2.1]heptane-2-carboxylic acid* (**II-12**). Yield 65%, white solid, mp: 188–192 °C; ^1^H-NMR δ: 1.44–1.69 (m, 4H, H-1, 2), 2.93–3.00 (m, 1H, H-4), 3.06 (d, *J* = 9.46 Hz, 1H, H-5), 4.66 (d, *J* = 4.41 Hz, 1H, H-6), 4.79 (d, *J* = 3.78 Hz, 1H, H-3), 7.44–7.49 (m, 2H, H-3′, 5′), 7.50-7.56 (m, 2H, H-2′, 6′), 9.85 (s, 1H, H-9), 12.01 (s, 1H, H-10); ^13^C-NMR δ: 28.89 (s, 1C, C-1), 29.45 (s, 1C, C-2), 52.16 (s, 1C, C-4), 53.88 (s, 1C, C-5), 77.36 (s, 1C, C-3), 79.07 (s, 1C, C-6), 114.90 (s, 1C, C-4′), 121.55 (s, 2C, C-2′, C-6′), 131.87 (s, 2C, C-3′, C-5′), 139.14 (s, 1C, C-1′), 169.98 (s, 1C, C-8), 172.66 (s, 1C, C-7); HR-MS (ESI): *m*/*z* calcd. For C_14_H_14_BrNO_4_Na ([M + Na]^+^) 362.0004, found 362.0015.

*3-((2-Iodophenyl)carbamoyl)-7-oxabicyclo[2.2.1]heptane-2-carboxylic acid* (**II-13**). Yield 74%, white solid, mp: 178–180 °C; ^1^H-NMR δ: 1.54–1.75 (m, 4H, H-1, 2), 3.12 (br, s, 2H, H-3, 6), 4.81 (br, s, 1H, H-6), 4.91 (br, s, 1H, H-3), 6.91 (t, *J* = 7.09 Hz, 1H, H-4′), 7.37 (t, *J* = 7.25 Hz, 1H, H-5′), 7.77 (d, *J* = 7.57 Hz, 1H, H-3′), 7.85 (d, *J* = 7.57 Hz, 1H, H-6′), 8.84 (br, s, 1H, H-9), 12.28 (br, s, 1H, H-10); ^13^C-NMR δ: 28.90 (1C, C-1), 29.02 (1C, C-2), 52.35 (1C, C-4), 54.66 (1C, C-5), 77.61 (1C, C-3), 79.33 (1C, C-6), 124.56 (1C, C-2′), 126.81 (1C, C-6′), 128.98 (1C, C-4′), 139.32 (1C, C-5′), 139.74 (1C, C-3′), 170.16 (1C, C-8), 172.58 (1C, C-7); HR-MS (ESI): *m*/*z* calcd. For C_14_H_14_INO_4_Na ([M + Na]^+^) 409.9865, found 409.9863.

*3-((3-Iodophenyl)carbamoyl)-7-oxabicyclo[2.2.1]heptane-2-carboxylic acid* (**II-14**). Yield 54%, white solid, mp: 174–175 °C; ^1^H-NMR δ: 1.41–1.71 (m, 4H, H-1, 2), 2.97 (d, *J* = 9.46 Hz, 1H, H-4), 3.07 (d, *J* = 9.46 Hz, 1H, H-5), 4.66 (d, *J* = 4.41 Hz, 1H, H-6), 4.80 (d, *J* = 3.78 Hz, 1H, H-3), 6.85 (td, *J* = 8.43, 2.36 Hz, 1H, H-4′), 7.22 (d, *J* = 8.20 Hz, 1H, H-6′), 7.29-7.36 (m, 1H, H-5′), 7.57 (d, *J* = 11.66 Hz, 1H, H-2′), 9.95 (s, 1H, H-9) 12.01 (s, 1H, H-10); ^13^C-NMR δ: 28.89 (s, 1C, C-1), 29.44 (s, 1C, C-2), 52.17 (s, 1C, C-4), 53.89 (s, 1C, C-5), 77.38 (s, 1C, C-3), 79.07 (s, 1C, C-6), 106.28 (s, 1C, C-4′), 109.72 (s, 1C, C-2′), 115.31 (s, 1C, C-6′), 130.62 (s, 1C, C-5′), 141.46 (s, 1C, C-1′), 161.65 (s, 1C, C-3′), 170.19 (s, 1C, C-8), 172.64 (s, 1C, C-7); HR-MS (ESI): *m*/*z* calcd. For C_14_H_14_INO_4_Na ([M + Na]^+^) 409.9865, 409.9871.

*3-((3-Iodophenyl)carbamoyl)-7-oxabicyclo[2.2.1]heptane-2-carboxylic acid* (**II-15**). Yield 81%, white solid, mp: 171–172 °C; ^1^H-NMR δ: 1.47–1.69 (m, 4H, H-1, 2), 2.96 (d, *J* = 9.46 Hz, 1H, H-4), 3.05 (d, *J* = 9.46 Hz, 1H, H-5), 4.65 (br, s, 1H, H-6), 4.79 (br, s, 1H, H-3), 7.39 (d, *J* = 8.51 Hz, 2H, H-3′, 5′), 7.62 (d, *J* = 8.51 Hz, 2H, H-2′, 6′), 9.81 (br, s, 1H, H-9), 12.01 (br, s, 1H, H-10); ^13^C-NMR δ: 28.90 (1C, C-1), 29.44 (1C, C-2), 52.19 (1C, C-4), 53.93 (1C, C-5), 77.38 (1C, C-3), 79.06 (1C, C-6), 86.66 (1C, C-4′), 121.86 (2C, C-2′, C-6′), 137.69 (2C, C-3′, C-5′), 139.61 (1C, C-1′), 169.98 (1C, C-8), 172.65 (1C, C-7); HR-MS (ESI): *m*/*z* calcd. For C_14_H_14_INO_4_Na ([M + Na]^+^) 409.9865, found 409.9871.

*3-((4-Hydroxyphenyl)carbamoyl)-7-oxabicyclo[2.2.1]heptane-2-carboxylic acid* (**II-16**). Yield 56%, white solid, mp: 174–175 °C; ^1^H-NMR δ: 1.42–1.74 (m, 4H, H-1, 2) 2.89–2.96 (m, 1H, H-4) 3.02 (d, *J* = 9.46 Hz, 1H, H-5) 4.62 (d, *J* = 3.78 Hz, 1H, H-6) 4.79 (d, *J* = 2.52 Hz, 1H, H-3) 6.68 (d, *J* = 8.20 Hz, 2H, H-3′, 5′) 7.30 (d, *J* = 8.51 Hz, 2H, H-2′, 6′) 9.13 (br, s, 1H, H-7′) 9.36 (s, 1H, H-9) 11.89 (br, s, 1H, H-10); ^13^C-NMR δ: 28.89 (s, 1C, C-1), 29.47 (s, 1C, C-2), 52.03 (s, 1C, C-4), 53.89 (s, 1C, C-5), 77.26 (s, 1C, C-3), 79.24 (s, 1C, C-6), 115.39 (s, 2C, C-3′, C-5′), 121.49 (s, 2C, C-2′, C-6′), 131.39 (s, 1C, C-1′), 153.62 (s, 1C, C-4′), 169.13 (s, 1C, C-8), 172.77 (s, 1C, C-7); HR-MS (ESI): *m*/*z* calcd for C_14_H_15_NO_5_Na ([M + Na]^+^) 300.0848, found 300.0851.

*3-((4-Carboxyphenyl)carbamoyl)-7-oxabicyclo[2.2.1]heptane-2-carboxylic acid* (**II-17**). Yield 44%, white solid, mp: 256–257 °C; ^1^H-NMR δ: 1.52–1.64 (m, 4H, H-1, 2), 2.98 (d, *J* = 9.46 Hz, 1H, H-4), 3.11 (d, *J* = 9.77 Hz, 1H, H-5), 4.68 (d, *J* = 4.41 Hz, 1H, H-6), 4.80 (d, *J* = 3.78 Hz, 1H, H-3), 7.66 (d, *J* = 8.83 Hz, 2H, H-2′, 6′), 7.89 (d, *J* = 8.83 Hz, 2H, H-3′, 5′), 10.05 (s, 1H, H-9), 12.35 (br, s, 2H, H-10); ^13^C-NMR δ: 28.91 (s, 1C, C-1) 29.44 (s, 1C, C-2) 52.20 (s, 1C, C-4) 53.97 (s, 1C, C-5) 77.42 (s, 1C, C-3) 79.11 (s, 1C, C-6) 118.80 (s, 2C, C-2′, C-6′) 125.29 (s, 1C, C-4′) 130.78 (s, 2C, C-3′, C-5′) 143.69 (m, 1C, C-1′) 167.32 (m, 1C, C-7′) 170.32 (s, 1C, C-8) 172.65 (s, 1C, C-7); HR-MS (ESI): *m*/*z* calcd for C_15_H_15_NO_6_Na ([M + Na]^+^) 328.0797, found 328.0796.

*3-((2-Methoxyphenyl)carbamoyl)-7-oxabicyclo[2.2.1]heptane-2-carboxylic acid* (**II-18**). Yield 66%white solid, mp: 151–152 °C; ^1^H-NMR δ: 1.52–1.72 (m, 4H, H-1, 2), 3.05–3.17 (m, 2H, H-4, 5), 3.83 (s, 3H, H-7′), 4.71 (d, *J* = 5.04 Hz, 1H, H-6′), 4.89 (d, *J* = 3.47 Hz, 1H, H-3′), 6.86–6.93 (m, 1H, H-5′), 7.02 (d, *J* = 4.10 Hz, 2H, H-3′, 4′), 8.11 (d, *J* = 7.88 Hz, 1H, H-6′), 8.87 (s, 1H, H-9), 12.20 (br, s, 1H, H-10); ^13^C-NMR δ: 28.96 (C-1, C-2), 52.44 (C-7′), 55.32 (C-4), 56.45 (C-5), 77.52 (C-3), 79.54 (C-6), 111.44 (C-3′), 120.32 (C-6′), 120.79 (C-5′), 123.84 (C-3′), 128.31 (C-4′), 148.84 (C-2′), 169.95 (C-8), 172.61 (C-7); HR-MS (ESI): *m*/*z* calcd for C_15_H_17_NO_5_Na ([M + Na]^+^) 314.1004, found 314.1008.

*3-((3-Methoxyphenyl)carbamoyl)-7-oxabicyclo[2.2.1]heptane-2-carboxylic acid* (**II-19**). Yield 73%, white solid, mp: 165–166 °C; ^1^H-NMR δ: 1.47–1.70 (m, 4H, H-1, 2), 2.95 (d, *J* = 9.46 Hz, 1H, H-4), 3.07 (d, *J* = 9.77 Hz, 1H, H-5), 4.64 (d, *J* = 4.41 Hz, 1H, H-6), 4.80 (d, *J* = 3.78 Hz, 1H, H-3), 6.61 (dd, *J* = 8.20, 1.89 Hz, 1H, H-4′), 7.05 (d, *J* = 8.20 Hz, 1H, H-6′), 7.15–7.22 (m, 1 H, H-5′), 7.29 (s, 1H, H-2′), 9.68 (s, 1H, H-9), 11.99 (br, s, 1H, H-10); ^13^C-NMR δ: 28.91 (C-2), 29.46 (C-1), 52.05 (C-7′), 54.03 (C-4), 55.42 (C-5), 77.34 (C-3), 79.23 (C-6), 105.36 (C-6′), 108.97 (C-2′), 111.89 (C-4′), 129.81 (C-5′), 140.95 (C-1′), 159.95 (C-3′), 169.83 (C-8), 172.72 (C-7); HR-MS (ESI): *m*/*z* calcd for C_15_H_17_NO_5_Na ([M + Na]^+^) 314.1004, found 314.0983.

*3-((4-Methoxyphenyl)carbamoyl)-7-oxabicyclo[2.2.1]heptane-2-carboxylic acid* (**II-20**). Yield 71%, white solid, mp: 167–168 °C; ^1^H-NMR: 1.46–1.69 (m, 4H, H-1, 2), 2.94 (d, *J* = 9.77 Hz, 1H, H-4), 3.03 (d, *J* = 9.46 Hz, 1H, H-5), 4.63 (d, *J* = 4.10 Hz, 1H, 1H, H-6), 4.79 (d, *J* = 3.78 Hz, 1H, 1H, H-3), 6.87 (d, *J* = 8.83 Hz, 2H, H-3′, 5′), 7.44 (d, *J* = 8.83 Hz, 2 H, H-2′, 6′), 9.51 (s, 1H, H-9), 11.93 (br, s, 1H, H-10); ^13^C-NMR δ: 28.89 (C-2), 29.47 (C-1), 52.07 (C-7′), 53.87 (C-4), 55.64 (C-5), 77.29 (C-3), 79.20 (C-6), 114.20 (C-3′, C-5′), 121.24 (C-2′, C-6′), 132.91 (C-1′), 155.54 (C-4′), 169.33 (C-8), 172.76 (C-7); HR-MS (ESI): *m*/*z* calcd for C_15_H_17_NO_5_Na ([M + Na]^+^) 314.1004, found 314.1025.

*3-((4-(Trifluoromethoxy)phenyl)carbamoyl)-7-oxabicyclo[2.2.1]heptane-2-carboxylic acid* (**II-21**). Yield 47%, yellow solid, mp: 155–156 °C; ^1^H-NMR δ: 1.46–1.69 (m, 4H, H-1, 2), 2.95–3.00 (m, 1H, H-4), 3.07 (d, *J* = 9.77 Hz, 1H, H-5), 4.67 (d, *J* = 4.41 Hz, 1H, H-6), 4.80 (d, *J* = 3.78 Hz, 1H, H-3), 7.30 (d, *J* = 8.83 Hz, 2H, H-3′, 5′), 7.65 (d, *J* = 8.83 Hz, 2H, H-2′, 6′), 9.91 (s, 1H, H-9), 12.01 (s, 1H, H-10); ^13^C-NMR δ: 28.84 (m, 1C, C-1), 29.44 (m, 1C, C-2), 52.17 (m, 1C, C-4), 53.89 (m, 1C, C-5), 77.34 (m, 1C, C-3), 79.07 (m, 1C, C-6), 120.88 (s, 2C, C-3′, C-5′), 121.65 (s, 1C, C-7′), 121.97 (s, 2C, C-2′, C-6′), 138.91 (m, 1C, C-1′), 143.80 (m, 1C, C-4′), 169.85 (m, 1C, C-8), 172.47 (m, 1C, C-7); HR-MS (ESI): *m*/*z* calcd for C_15_H_14_F_3_NO_5_Na ([M + Na]^+^) 368.0722, found 368.0732.

*3-((4-Cyanophenyl)carbamoyl)-7-oxabicyclo[2.2.1]heptane-2-carboxylic acid* (**II-22**). Yield, 73%, yellow solid, mp: 164–166 °C; ^1^H-NMR δ: 1.46–1.70 (m, 4H, H-1, 2), 3.00 (d, *J* = 9.46 Hz, 1H, H-4), 3.09 (d, *J* = 9.77 Hz, 1H, H-5), 4.69 (d, *J* = 2.52 Hz, 1H, H-6), 4.80 (br, s, 1H, H-3), 7.74 (s, 2H, H-3′, 5′), 7.77–7.79 (m, 2H, H-2′, 6′), 10.20 (s, 1H, H-9), 12.05 (s, 1H, H-10); ^13^C-NMR δ: 28.91 (s, 1C, C-1) 29.42 (s, 1C, C-2) 52.33 (s, 1C, C-4) 53.88 (m, 1C, C-5) 77.49 (s, 1C, C-3) 78.97 (s, 1C, C-6) 105.05 (s, 1C, C-4′) 119.56 (s, 1C, C-7′) 133.65 (s, 2C, C-2′, C-6′) 143.99 (s, 2C, C-3′, C-5′) 170.62 (s, 1C, C-8) 172.57 (s, 1C, C-7); HR-MS (ESI): *m*/*z* calcd for C_15_H_14_N_2_O_4_Na ([M + Na]^+^) 309.0851,found 309.0856.

*3-((2-Nitrophenyl)carbamoyl)-7-oxabicyclo[2.2.1]heptane-2-carboxylic acid* (**II-23**). Yield, 47%, yellow solid, mp: 155–156 °C; ^1^H-NMR δ: 1.46–1.72 (m, 4H, H-1, 2), 2.98–3.12 (m, 2H, H-4, 5), 4.63–4.85 (m, 2H, H-3, 6), 7.60 (t, *J* = 8.20 Hz, 1H, H-4′), 7.82 (d, *J* = 7.88 Hz, 1H, H-5′), 7.90 (d, *J* = 7.88 Hz, 1H, H-6′), 8.65 (br, s, 1H, H-3′), 10.28 (br, s, 1H, H-9), 12.02-12.09 (m, 1H, H-10); ^13^C-NMR δ: 28.91 (C-2), 29.44 (C-1), 52.24 (C-4), 53.82 (C-5), 77.49 (C-3), 79.00 (C-6), 113.68 (C-6′), 117.96 (C-4′), 125.55 (C-3′), 130.53 (C-5′), 140.91 (C-1′), 148.42 (C-2′), 170.61 (C-8), 172.63 (C-7); HR-MS (ESI): *m*/*z* calcd for C_14_H_14_N_2_O_6_Na ([M + Na]^+^) 329.0750, found 329.0754.

*3-((3-Nitrophenyl)carbamoyl)-7-oxabicyclo[2.2.1]heptane-2-carboxylic acid* (**II-24**). Yield, 44%, yellow solid, mp: 178–179 °C; ^1^H-NMR δ: 1.45–1.70 (m, 4H, H-1, 2), 2.94–3.01 (m, 1H, H-4), 3.03–3.09 (m, 1H, H-5), 4.67 (br, s, 1H, H-6), 4.79 (br, s, 1H, H-3), 7.19–7.30 (m, 2H, H-4′, 6′), 7.39 (d, J = 7.88 Hz, 1H, H-5′), 7.96 (br, s, 1H, H-2′), 9.91 (br, s, 1H, H-9) 12.01 (s, 1H, H-10); ^13^C-NMR δ: 28.54 (C-2), 29.36 (C-1), 52.44 (C-4), 53.79 (C-5), 77.80 (C-3), 79.55 (C-6), 115.24 (C-2′), 119.30 (C-4′), 126.72 (C-6′), 130.81 (C-5′), 140.68 (C-1′), 149.11 (C-3′); HR-MS (ESI): *m*/*z* calcd for C_14_H_14_N_2_O_6_Na ([M + Na]^+^) 329.0750, found 329.0771.

*3-((4-Nitrophenyl)carbamoyl)-7-oxabicyclo[2.2.1]heptane-2-carboxylic acid* (**II-25**). Yield, 54%, yellow solid, mp: 156–157 °C; ^1^H-NMR δ: 1.48–1.70 (m, 4H, H-1, 2), 3.02 (d, *J* = 9.46 Hz, 1H, H-4), 3.12 (d, *J* = 9.46 Hz, 1H, H-5), 4.71 (br, s, 1H, H-6), 4.81 (br, s, 1H, H-3), 7.80 (d, *J* = 8.83 Hz, 2H, H-2′, 6′), 8.22 (d, *J* = 8.83 Hz, 2H, H-3′, 5′), 10.40 (s, 1H, H-9), 12.09 (br, s, 1H, H-10); ^13^C-NMR δ: 28.92 (C-2), 29.42 (C-1), 52.39 (C-4), 53.89 (C-5), 77.56 (C-3), 78.96 (C-6), 119.15 (C-2′, C-6′), 125.40 (C-3′, C-5′), 142.41 (C-4′), 146.01 (C-1′), 170.79 (C-8), 172.56 (C-7); HR-MS (ESI): *m*/*z* calcd for C_14_H_14_N_2_O_6_Na ([M + Na]^+^) 329.0750, found 329.0763.

*3-((3,4-Dichlorophenyl)carbamoyl)-7-oxabicyclo[2.2.1]heptane-2-carboxylic acid* (**II-26**). Yield, 88%, white solid, mp: 160–161 °C; ^1^H-NMR δ: 1.45–1.69 (m, 4H, H-1, 2), 2.95–3.01 (m, 1H, H-4), 3.02–3.08 (m, 1H, H-5), 4.67 (d, *J* = 3.78 Hz, 1H, H-6), 4.79 (d, *J* = 2.84 Hz, 1H, H-3), 7.40 (d, *J* = 8.83 Hz, 1H, H-5′), 7.55 (d, *J* = 8.83 Hz, 1H, H-6′), 7.99 (s, 1H, H-2′), 10.06 (s, 1H, H-9), 12.04 (s, 1H, H-10); ^13^C-NMR δ: 28.86 (m, 1C), 29.43 (s, 1C), 52.26 (s, 1C), 53.78 (s, 1C), 77.42 (s, 1C), 78.95 (s, 1C), 119.64 (s, 1C), 120.86 (s, 1C), 124.77 (s, 1C), 131.02 (s, 1C), 131.32 (s, 1C), 139.80 (s, 1C), 170.29 (s, 1C), 172.61 (s, 1C); HR-MS (ESI): *m*/*z* calcd for C_14_H_13_C_l2_NO_4_Na ([M + Na]^+^) 352.0119, found 352.0113.

*3-((2,4-Dibromophenyl)carbamoyl)-7-oxabicyclo[2.2.1]heptane-2-carboxylic acid* (**II-27**). Yield, 18%, white solid, mp: 241–242 °C; ^1^H-NMR δ: 1.59–1.68 (m, 4H, H-1, 2), 3.14 (s, 2H, H-4, 5), 4.79 (d, *J* = 4.10 Hz, 1H, H-6), 4.91 (br, s, 1H, H-3), 7.58 (d, *J* = 8.83 Hz, 1H, H-5′), 7.88 (s, 1H, H-6′), 7.95 (d, *J* = 8.83 Hz, 1H, H-3′), 9.06 (s, 1H, H-9), 12.31 (br, s, 1H, H-10); ^13^C-NMR δ: 28.77 (1C, C-1), 28.99 (1C, C-2), 52.46 (1C, C-4), 54.78 (1C, C-5), 77.72 (1C, C-3), 79.37 (1C, C-6), 115.70 (1C, C-4′), 116.51 (1C, C-2′), 125.09 (1C, C-6′), 131.40 (1C, C-3′), 134.66 (1C, C-5′), 136.36 (1C, C-1′), 170.37 (1C, C-8), 172.56 (1C, C-7); HR-MS (ESI): *m*/*z* calcd for C_14_H_13_Br_2_NO_4_Na ([M + Na]^+^) 441.9089, found 441.9096.

*3-((4-Iodo-2-methylphenyl)carbamoyl)-7-oxabicyclo[2.2.1]heptane-2-carboxylic acid* (**II-28**). Yield, 56%, white solid, mp: 147–149 °C; ^1^H-NMR δ: 1.59 (br, s, 4H, H-1, 2), 2.26 (s, 3H, H-7′), 3.04–3.10 (m, 1H, H-4), 3.12–3.18 (m, 1H, H-5), 4.74–4.79 (m, 1H, H-6), 4.81–4.87 (m, 1H, H-3), 7.42 (s, 1H, H-5′), 7.67 (d, *J* = 8.20 Hz, 1H, H-6′), 7.80 (d, *J* = 7.88 Hz, 1H, H-4′), 9.29–9.35 (m, 1H, H-9), 12.17–12.21 (m, 1H, H-10); ^13^C-NMR δ: 13.76 (1C, C-7′), 28.91 (1C, C-1), 29.22 (1C, C-2), 52.33 (1C, C-4), 53.72 (1C, C-5), 77.57 (1C, C-3), 79.13 (1C, C-6), 120.49 (1C, C-4′), 125.27 (1C, C-6′), 126.95 (1C, C-2′), 129.20 (1C, C-5′), 128.61 (1C, C-1′), 151.26 (1C, C-3′), 170.18 (1C, C-8), 172.59 (1C,C-7); HR-MS (ESI): *m*/*z* calcd for C_15_H_16_INO_4_Na ([M + Na]^+^) 424.0022, found 424.0025.

*3-((3,5-Dimethoxyphenyl)carbamoyl)-7-oxabicyclo[2.2.1]heptane-2-carboxylic acid* (**II-29**). Yield, 74%, white solid, mp: 156–157 °C; ^1^H-NMR δ: 1.47–1.68 (m, 4H, H-1, 2), 2.94 (d, *J* = 9.77 Hz, 1H, H-4), 3.05 (d, *J* = 9.46 Hz, 1H, H-5), 3.71 (s, 6H, H-7′, 8′), 4.63 (d, *J* = 3.47 Hz, 1H, H-6), 4.79 (br, s, 1H, H-3), 6.20 (br, s, 1H, H-4′), 6.80 (s, 2H, H-2′, 6′), 9.67 (s, 1H, H-9), 11.98 (br, s, 1H, H-10); ^13^C-NMR δ: 28.91 (1C, C-1), 29.42 (1C, C-2), 40.02 (2C, C-7′, C-8′), 52.33 (1C, C-4), 53.89 (1C, C-5), 77.49 (1C, C-3), 78.97 (1C, C-6), 105.05 (1C, C-4′), 119.56 (2C, C-2′, C-6′), 133.65 (1C, C-1′), 143.99 (2C, C-3′, C-5′), 170.62 (1C, C-8), 172.57 (1C, C-7); HR-MS (ESI): *m*/*z* calcd for C_16_H_19_NO_6_Na ([M + Na]^+^) 344.1110, found 344.1111.

*3-((2,5-Dimethoxyphenyl)carbamoyl)-7-oxabicyclo[2.2.1]heptane-2-carboxylic acid* (**II-30**). Yield 64%, white solid, mp: 188–189 °C; ^1^H-NMR δ: 1.58 (br, s, 4H, H-1, 2), 2.97–3.23 (m, 2H, H-4, 5), 3.55–3.94 (m, 6H, H-7′, 8′), 4.71 (br, s, 1H, H-6), 4.89 (br, s, 1H, H-3), 6.58 (br, s, 1H, H-4′), 6.94 (br, s, 1H, H-3′), 7.83 (br, s, 1H, H-6′), 8.89 (br, s, 1H, H-9), 12.10–12.40 (m, 1H, H-10); ^13^C-NMR δ: 28.8 (1C, C-1), 28.96 (1C, C-2), 52.46 (1C, C-4), 55.33 (1C, C-5), 55.80 (1C, C-8′), 57.11 (1C, C-7′), 77.57 (1C, C-3), 79.50 (1C, C-6), 107.14 (1C, C-6′), 107.58 (1C, C-3′), 112.29 (1C, C-4′), 129.20 (1C, C-1′), 143.25 (1C, C-2′), 153.64 (1C, C-5′), 170.11 (1C, C-8), 172.59 (1C, C-7); HR-MS (ESI): *m*/*z* calcd for C_16_H_19_NO_6_Na ([M + Na]^+^) 344.1110, found 344.1119.

*3-((2-Methyl-3-nitrophenyl)carbamoyl)-7-oxabicyclo[2.2.1]heptane-2-carboxylic acid* (**II-31**). Yield, 35%, white solid, mp: 144–145 °C; ^1^H-NMR δ: 1.59 (br, s, 4H, H-1, 2), 2.26 (s, 3H, H-7′), 3.04–3.10 (m, 1H, H-4), 3.12–3.18 (m, 1H, H-5), 4.74–4.79 (m, 1H, H-6), 4.81–4.87 (m, 1H, H-3), 7.42 (s, 1H, H-5′), 7.67 (d, *J* = 8.20 Hz, 1H, H-6′), 7.80 (d, *J* = 7.88 Hz, 1H, H-4′), 9.29–9.35 (m, 1H, H-9), 12.17–12.21 (m, 1H, H-10); ^13^C-NMR δ: 13.76 (1C, C-7′), 28.91 (1C, C-1), 29.22 (1C, C-2), 52.33 (1C, C-4), 53.72 (1C, C-5), 77.57 (1C, C-3), 79.13 (1C, C-6), 120.49 (1C, C-4′), 125.27 (1C, C-6′), 126.95 (1C, C-2′), 129.20 (1C, C-5′), 128.61 (1C, C-1′), 151.26 (1C, C-3′), 170.18 (1C, C-8), 172.59 (1C,C-7); HR-MS (ESI): *m*/*z* calcd for C_15_H_16_N_2_O_6_Na ([M + Na]^+^) 343.0906, found 343.0911.

*3-(Naphthalen-1-ylcarbamoyl)-7-oxabicyclo[2.2.1]heptane-2-carboxylic acid* (**II-32**). Yield, 69%, white solid, mp: 187–188 °C; ^1^H-NMR δ: 1.62 (br, s, 4H, H-1, 2), 3.11 (d, *J* = 9.77 Hz, 1H, H-4), 3.29 (s, 1H, H-5), 4.79-4.85 (m, 1H, H-6), 4.86–4.91 (m, 1H, H-3), 7.49 (s, 1H, H-2′), 7.53–7.61 (m, 2H, H-3′, 9′), 7.73 (s, 1H, H-8′), 7.78–7.84 (m, 1H, H-4′), 7.91–7.96 (m, 1H, H-7′), 8.03–8.08 (m, 1H, H-10′), 9.57 (s, 1H, H-9), 12.18 (s, 1H, H-10); ^13^C-NMR δ: 28.97 (1C, C-1), 29.28 (1C, C-2), 52.36 (1C, C-4), 54.14 (1C, C-5), 77.60 (1C, C-3), 79.49 (1C, C-6), 120.54 (1C, C-2′), 122.63 (1C, C-4′), 125.01 (1C, C-7′), 125.99 (1C, C-6′), 126.28 (1C, C-8′), 126.43 (1C, C-9′), 127.47 (1C, C-3′), 128.61 (1C, C-10′), 134.04 (1C, C-5′), 134.09 (1C, C-1′), 170.35 (1C, C-8), 172.86 (1C, C-7); HR-MS (ESI): *m*/*z* calcd for C_18_H_17_NO_4_Na ([M + Na]^+^) 334.1055, found 334.1056.

*3-(Pyridin-2-ylcarbamoyl)-7-oxabicyclo[2.2.1]heptane-2-carboxylic acid* (**II-33**). Yield, 46%, white solid, mp: 178–180 °C; ^1^H-NMR δ: 1.46–1.68 (m, 4H, H-1, 2), 3.02 (d, *J* = 9.46 Hz, 1H, H-4), 3.21 (d, *J* = 9.46 Hz, 1H, H-5), 4.69 (d, *J* = 5.04 Hz, 1H, H-6), 4.83 (d, *J* = 3.78 Hz, 1H, H-3), 7.33–7.40 (m, 1H, H-4′), 7.76 (t, *J* = 7.72 Hz, 1H, H-5′), 7.89 (d, *J* = 4.41 Hz, 1H, H-6′), 8.03 (d, *J* = 8.20 Hz, 1H, H-3′), 9.97 (s, 1H, H-9); ^13^C-NMR δ: 28.90 (1C, C-1), 29.24 (1C, C-2), 52.28 (1C, C-4), 53.92 (1C, C-5), 77.54 (1C, C-3), 79.28 (1C, C-6), 119.65 (1C, C-6′), 137.48 (1C, C-4′), 147.97 (1C, C-5′), 152.43 (1C, C-3′), 160.07 (1C, C-1′), 170.60 (1C, C-8), 172.74 (1C, C-7); HR-MS (ESI): *m*/*z* calcd for C_13_H_15_N_2_O_4_ ([M + 1] ^+^) 263.1135, found 263.1033.

*3-(Thiazol-2-ylcarbamoyl)-7-oxabicyclo[2.2.1]heptane-2-carboxylic acid* (**II-34**). Yield, 56%, white solid, mp: 174–176 °C; ^1^H-NMR δ: 1.42–1.70 (m, 4H, H-1, 2), 3.02 (d, *J* = 9.46 Hz, 1H, H-4), 3.22 (d, *J* = 9.46 Hz, 1H, H-5), 4.67 (d, *J* = 4.10 Hz, 1H, H-6), 4.80 (d, *J* = 3.15 Hz, 1H, H-3), 7.18 (d, *J* = 3.47 Hz, 1H, H-5′), 7.45 (d, *J* = 3.15 Hz, 1H, H-4), 11.83–11.98 (m, 1H, H-9), 12.00–12.18 (m, 1H, H-10); ^13^C-NMR δ: 28.85 (1C, C-1), 29.42 (1C, C-2), 52.30 (1C, C-4), 52.43 (1C, C-5), 77.61 (1C, C-3), 79.05 (1C, C-6), 113.62 (1C, C-5′), 137.92 (1C, C-4′), 158.54 (1C, C-1′), 169.81 (1C, C-8), 172.45 (1C, C-7); HR-MS (ESI): *m*/*z* calcd for C_11_H_12_N_2_O_4_SNa ([M + Na]^+^) 291.0415, found 291.0411.

*3-(Benzo[d]thiazol-2-ylcarbamoyl)-7-oxabicyclo[2.2.1]heptane-2-carboxylic acid* (**II-35**). Yield, 89%, white solid, mp: 175–176 °C; ^1^H-NMR: 1.45–1.73 (m, 4H, H-1, 2), 3.09 (d, *J* = 9.14 Hz, 1H, H-4), 3.24 (br, s, 1H, H-5), 4.75 (br, s, 1H, H-6), 4.82 (br, s, 1H, H-3), 7.26–7.47 (m, 2H, H-7′, 8′), 7.70–7.77 (m, 1H, H-6′), 7.94-8.02 (m, 1H, H-9′), 12.19 (br, s, 2H, H-9, 10); ^13^C-NMR δ: 28.87 (1C, C-1), 29.38 (1C, C-2), 52.55 (1C, C-4), 52.63 (1C, C-5), 77.84 (1C, C-3), 78.86 (1C, C-6), 120.84 (1C, C-9′), 122.07 (1C, C-6′), 123.77 (1C, C-7′), 126.46 (1C, C-8′), 131.84 (1C, C-3′), 149.01 (1C, C-4′), 158.52 (1C, C-1′), 170.82 (1C, C-8), 172.31 (1C, C-7); HR-MS (ESI): *m*/*z* calcd for C_15_H_14_N_2_O_4_SNa ([M + Na]^+^) 341.0572, found 341.0579.

*3-((1H-Benzo[d]imidazol-2-yl)carbamoyl)-7-oxabicyclo[2.2.1]heptane-2-carboxylic acid* (**II-36**). Yield, 45%, white solid, mp: 189–190 °C; ^1^H-NMR δ: 1.42–1.70 (m, 4H, H-1, 2), 3.02 (d, J = 9.46 Hz, 1H, H-4), 3.22 (d, J = 9.46 Hz, 1H, H-5), 4.67 (d, J = 4.10 Hz, 1H, H-6), 4.80 (d, J = 3.15 Hz, 1H, H-3), 7.28 (dd, J = 5.36, 2.84 Hz, 2H, H-6′, 9′), 7.61 (br, s, 2H, H-7′, 8′), 10.15 (s, 1H, H-9), 12.05 (s, 1H, H-10); ^13^C-NMR δ: 28.90 (1C, C-1), 29.47 (1C, C-2), 52.11 (1C, C-4), 53.88 (1C, C-5), 77.33 (1C, C-3), 79.20 (1C, C-6), 111.90 (2C, C-6′, C-9′), 119.73 (2C, C-7′, C-8′), 138.96 (2C, C-3′, C-4′), 155.36 (1C, C-1′), 176.17 (2C, C-7, C-8); HR-MS (ESI): *m/z* calcd for C_15_H_15_N_3_O_4_Na ([M + Na]^+^) 324.0960, found 324.0963.

### 3.3. Screening of Antifungal Activity in Vitro

The antifungal activity of the synthetic compounds *in vitro* against eight plant pathogenic fungi (*Valsa mali*, *B. berengeriana*, *S. fructigena*, *G. cingulate*, *A. alternate*, *S. sclerotiorum*, *A. solani* and *C. sativum*) was assayed by the mycelium growth rate method with slight modification [27]. All of the fungi were provided by Laboratory of Integrated Management of Plant Diseases, Northwest A & F University (Yangling, China). The isolates were cultured for 5 days at 25 ± 1 °C on potato dextrose agar (PDA).

Antifungal activity was assessed as follows: the synthesized compounds were screened *in vitro* for their antifungal activities against the eight phytopathogenic fungi. PDA medium was prepared in the flasks and sterilized. Those compounds were dissolved in DMSO at a concentration of 50 μg/mL. DMSO served as the control, while commercially available agricultural fungicide thiabendazole was used as a positive control for its high efficiency and broad spectrum of antifungal activity. Each sample was measured in three replicates, each colony diameter of the three replicates was measured four times by a cross bracketing method. After the mycelia completed growth, the diameters of the mycelial masses were measured and the inhibition rates were calculated according to the following formula and expressed as means ± S.D.:

Growth inhibition rate (%) = [(*d_c_* − *d_0_*) − (*d_t_* − *d*_0_)]/(*d_c_* − *d*_0_) × 100 (1)

where *d*_0_: diameter of the fungus cut-outs, *d_c_*: average diameter of the untreated control fungus, and *d_t_* is the average diameter of mycelia on treated PDA with those compounds.

Based on the results of preliminary screening, the final stock solutions of the tested compounds dissolved in acetone were 100, 50, 25, 10, and 5 μg/mL. The medium was then poured into sterilized Petri dishes. All types of fungi were incubated in PDA at 25 ± 1 °C for 5 days to get new mycelium for the antifungal assays, and a mycelia disk of approximately 5 mm diameter cut-out from culture medium was picked up with a sterilized inoculation needle and inoculated in the center of the PDA Petri dishes with different concentrations of NCTD derivatives [29,30]. The inoculated Petri dishes were incubated at 25 ± 1 °C for 4 days. The IC_50_ (median inhibitory concentration) values of some compounds were determined, and the results are listed in Table 2.

## 4. Conclusions

In summary, we have reported the synthesis of a series of NCTD derivatives with aromatic amine moieties as well as the ability of these compounds to inhibit the growth of eight fungal phytopathogens. Seven of these synthetic compounds presented significant fungistatic activities against all of the eight fungi, superior to the corresponding parent compound NCTD for some fungi, and in some cases they were the same as or more active than TBZ. Compound **II-8** exhibited the most significant activity on all eight fungi, much better than TBZ, NCTD and CTD. In particular, **II-8** showed excellent antifungal properties against *S. fructigena* and *S. sclerotiorum*, with IC_50_ values of 0.88 and 0.97 μg/mL, respectively. SAR data for these compounds are as follows: (1) the benzene ring is critical for the improvement of the spectrum of antifungal activity and the inhibition of *B. berengeriana*, *G. cingulate*, *A. alternate*, *S. sclerotiorum*, *A. solani* and *C. sativum* (c.f. **II-1** *vs.* norcantharidin and cantharidin); (2) among the three sites, including the C-2′, C-3′ and C-4′ positions of the phenyl ring, the presence of the halogen atom at the C-3′ position of the phenyl ring caused the most significant increase in antifungal activity (**II-5** *vs.* **II-4** and **II-6**, **II-8** *vs.* **II-7** and **II-9**, **II-11** *vs.* **II-10** and **II-12**); (3) compounds with substitutions of strongly electron-drawing or electron-donating groups were found to have a poor antifungal activity; and (4) compared with fluorine, bromine and iodine, one chlorine atom substituted at C-3′ position of the benzene ring gave the highest fungistatic activity (**II-8** *vs.* **II-5**, **II-11** and **II-14**). Taken together, the data demonstrated that compound **II-8** possesses the most potent inhibitory activity toward the fungal plant pathogens tested in this study and could be a potential lead structure for further discovery of novel antifungal agrochemicals.
